# Assessing the diversity and distribution of potential intermediate hosts snails for urogenital schistosomiasis: *Bulinus* spp. (Gastropoda: Planorbidae) of Lake Victoria

**DOI:** 10.1186/s13071-020-04281-1

**Published:** 2020-08-14

**Authors:** Fred D. Chibwana, Immaculate Tumwebaze, Anna Mahulu, Arthur F. Sands, Christian Albrecht

**Affiliations:** 1 Department of Animal Ecology and Systematics, Justus Liebig University Giessen, Giessen, Germany; 2 Department of Zoology and Wildlife Conservation, University of Dar es Salaam, Dar es Salaam, Tanzania

**Keywords:** African lakes, Epidemiology, Phylogeography, Neglected tropical diseases, *Schistosoma haematobium*

## Abstract

**Background:**

The Lake Victoria basin is one of the most persistent hotspots of schistosomiasis in Africa, the intestinal form of the disease being studied more often than the urogenital form. Most schistosomiasis studies have been directed to *Schistosoma mansoni* and their corresponding intermediate snail hosts of the genus *Biomphalaria*, while neglecting *S. haematobium* and their intermediate snail hosts of the genus *Bulinus*. In the present study, we used DNA sequences from part of the cytochrome *c* oxidase subunit 1 (*cox*1) gene and the internal transcribed spacer 2 (ITS2) region to investigate *Bulinus* populations obtained from a longitudinal survey in Lake Victoria and neighbouring systems during 2010–2019.

**Methods:**

Sequences were obtained to (i) determine specimen identities, diversity and phylogenetic positions, (ii) reconstruct phylogeographical affinities, and (iii) determine the population structure to discuss the results and their implications for the transmission and epidemiology of urogenital schistosomiasis in Lake Victoria.

**Results:**

Phylogenies, species delimitation methods (SDMs) and statistical parsimony networks revealed the presence of two main groups of *Bulinus* species occurring in Lake Victoria; *B. truncatus*/*B. tropicus* complex with three species (*B. truncatus*, *B. tropicus* and *Bulinus* sp. 1), dominating the lake proper, and a *B. africanus* group, prevalent in banks and marshes. Although a total of 47 *cox*1 haplotypes, were detected within and outside Lake Victoria, there was limited haplotype sharing (only Haplotype 6 was shared between populations from Lake Victoria open waters and neighbouring aquatic systems) – an indication that haplotypes are specific to habitats.

**Conclusions:**

The *Bulinus* fauna of Lake Victoria consists of at least *B. truncatus*, *B. tropicus*, *Bulinus* sp. 1 (*B. trigonus*?) and *B. ugandae*. The occurrence and wide distribution of *Bulinus* species in Lake Victoria potentially implies the occurrence of urogenital schistosomiasis in communities living along the shores and on islands of the lake who depend solely on the lake for their livelihood. More in-depth studies are needed to obtain a better picture of the extent of the disease in the Lake Victoria basin. 
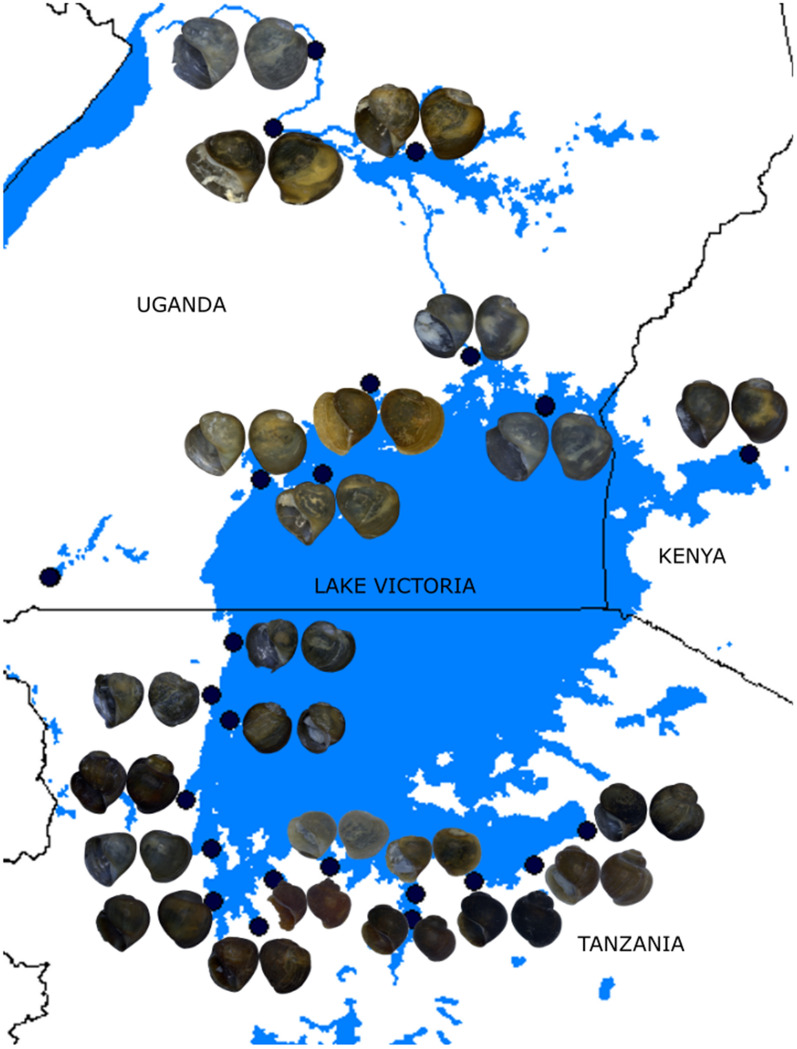

## Background

Schistosomiasis is a parasitic disease caused by digenean trematodes of the genus *Schistosoma* and is a socio-notable disease in tropical and subtropical regions. It is prevalent in more than 78 countries and territories infecting more than 250 million people worldwide, most of whom inhabit sub-Saharan Africa [1, 2]. Although more than 20 *Schistosoma* species are recognised, only *Schistosoma mansoni* and *S. haematobium* are ubiquitously known in sub-Saharan Africa due to their capability to cause intestinal and urogenital schistosomiasis, respectively [1, 3, 4]. The highest infections and disease burdens are frequently found in school-aged children, particularly in settings with poor hygiene and sanitary facilities [5]. Human hosts infected with these *Schistosoma* species experience acute hyperaemia, abnormal growth, internal haemorrhaging, fibrosis and tissue thickening [6]. As a result, infection with *S. mansoni* culminates with liver fibrosis, portal hypertension and ascites, while bladder cancer is the final stage of a *S. haematobium* infection [7]. Furthermore, genital schistosomiasis complications associated with *S. haematobium* infections include hypertrophic and ulcerative lesions of the female genital tract [8]. *Schistosoma* species, like other digenean trematodes, utilise pulmonate snails to complete their two-host life-cycles; i.e. *Biomphalaria* spp. for *S. mansoni* and *Bulinus* spp. for *S. haematobium* [1, 3, 4].

The Lake Victoria ecoregion of the East African Rift System, is characterized by a wealth of extraordinary freshwater biodiversity that has accumulated throughout the Quaternary, including almost 700 species of cichlid fishes [9, 10]. Major geological and climatological changes occurred in this region during this period. These changes are linked to the development of the East African Rift. More recently, anthropogenic pressures in the Lake Victoria ecoregion have grown exponentially due to multifactorial stressors such as habitat degradation, pollution, exploitation, the introduction of invasive species, ecosystem modifications and climate change [11, 12]. Insights into the consequences of recent and historic environmental changes in the region are crucial to understanding the diversification dynamics of freshwater biota. Effects of ecosystem changes on the community composition and demography of benthic organisms remain poorly assessed since few studies have been conducted apart from cichlid fishes and the schistosome intermediate host snail genus *Biomphalaria* [13].

Lake Victoria is endowed with a remarkable mollusc fauna, although it is less diverse than in lakes Malawi and Tanganyika, perhaps due to its younger age and relative shallowness [10, 14]. Despite its young age of about 400,000 years, Lake Victoria has experienced three major desiccation events within the last 100,000 years [15, 16]. The current water body arose about 14,600 years ago [15, 16], which is relatively shorter for snail species radiation [10, 17]. Nevertheless, Brown et al. [17] listed 28 gastropod species in Lake Victoria, of which six are medically significant species within genera *Bulinus* (4 species) and *Biomphalaria* (2 species). Lake Victoria, which is shared between Tanzania, Uganda and Kenya, therefore plays a significant role in the persistence of schistosomiasis in these surrounding countries [18–20]. Despite the increasing efforts to control schistosomiasis with praziquantel through mass drug administration (MDA) programmes, East African countries are still among the hotspots for this parasitic disease. Herein, the majority of schistosomiasis cases are reported from fishing communities and particularly in school-aged children surrounding Lake Victoria [21–24]. The vast majority of studies focusing on *Schistosoma* and their intermediate hosts in Lake Victoria and neighbouring aquatic systems have mainly focused on *S. mansoni* and *Biomphalaria* spp. [13, 18, 25] while overlooking *Bulinus* spp. and their potential role in the urogenital schistosomiasis transmission (i.e. *S. haematobium*). However, identifying these potential *Bulinus* hosts is an initial step in estimating the extent and relevance of urogenital schistosomiasis in the given area [26, 27].

The genus *Bulinus* consists of 37 species occurring mainly in Africa, the Middle East and in the Mediterranean Area [17]. The recognized *Bulinus* species fall into four groups, namely the *Bulinus africanus*, *B. reticulatus*, and *B. forskalii* species groups, and the *B. truncatus*/*B. tropicus* species complex. Many species within these groups except, for example, *B. tropicus* and *B. ugandae* are involved in the transmission of *S. haematobium* [28, 29]. Moreover, *B. africanus* group species play an important role in the transmission of *S. haematobium* and *S. bovis* in Central East Africa [17]. However, precise species identification of snails of the genus *Bulinus* is often difficult because of strong morphological similarities and overlap among species, the coexistence of different forms and groups in a narrow area and the lack of well-defined criteria by which to distinguish species [17, 29]. Additionally, some studies have also reported the existence of cryptic species in some localities [30, 31], which further exacerbates the taxonomic uncertainties within genus *Bulinus*.

The knowledge of the number of *Bulinus* species occurring within or nearby Lake Victoria is obscure. For instance, Mandahl-Barth [29] recognised *B. trigonus* and *B. transversalis* as independent species, but Brown [17] viewed them as lacustrine morphs of *B. tropicus* and *B. truncatus* or synonyms of unnamed *Bulinus* species (*Bulinus* sp.). Moreover, there is a scarcity of information on the geographical distribution patterns of *Bulinus* species in the lake. *Bulinus trigonus* and *B. transversalis* have their type-localities in the Tanzanian side of Lake Victoria, while *B. ugandae* was first found in Jinja Bay, Uganda [17]. Surveys by Mwambungu [32] reported the occurrence of *B. ugandae* in the Speke Gulf of the lake in Tanzania, Ngupula & Kayanda [33] found *B. ugandae* and *B. transversalis* in Uganda and Opisa et al. [34] and Nyakaana et al. [35] reported the existence of *B. globosus* in the lake shores in Kenya and Uganda. Although the separation of *B. ugandae* from *B. globosus* is dubious and the overall taxonomy of *Bulinus* spp. in Lake Victoria is uncertain [17], it is not clear if all the four *Bulinus* groups are represented in the lake. Moreover, knowledge of how the four groups may be spatially distributed remains questionable. Moreover, *Bulinus* species such as *B. ugandae* and *B. trigonus*, whose type-materials come from Lake Victoria, are not endemic to the lake, similar morphs have been recorded in lakes Mutanda and Edward as well [17].

In many biological cases where conventional analyses have failed to identify species, molecular techniques, particularly phylogenetic approaches using DNA sequence data, have proven successful. For example, the application of markers, such as cytochrome *c* oxidase subunit 1 (*cox*1) and nuclear genes such as the internal transcribed spacer (ITS) regions, *28S* and *18S*, have facilitated species identification of *Bulinus* spp. [31, 36, 37]. In the present study we, therefore, used two more variable genetic markers, *cox*1 and ITS2, to investigate the phylogeography of *Bulinus* species occurring in Lake Victoria. This information is invaluable in improving our understanding of *Bulinus* species identities and phylogenetic relationships, as well as the epidemiology of the potential urogenital schistosomiasis.

Therefore, we combine mitochondrial DNA (mtDNA) and nuclear DNA (nDNA) markers to investigate *Bulinus* populations obtained from a longitudinal survey in Lake Victoria and neighbouring aquatic systems to (i) determine the identity, diversity and phylogenetic position of the species, (ii) reconstruct phylogeographical affinities and (iii) determine the population structures of the species. We discuss the results and their implications for the potential transmission and epidemiology of urogenital schistosomiasis in Lake Victoria.

## Methods

### Source of material for genomic DNA

**Fig. 1:**
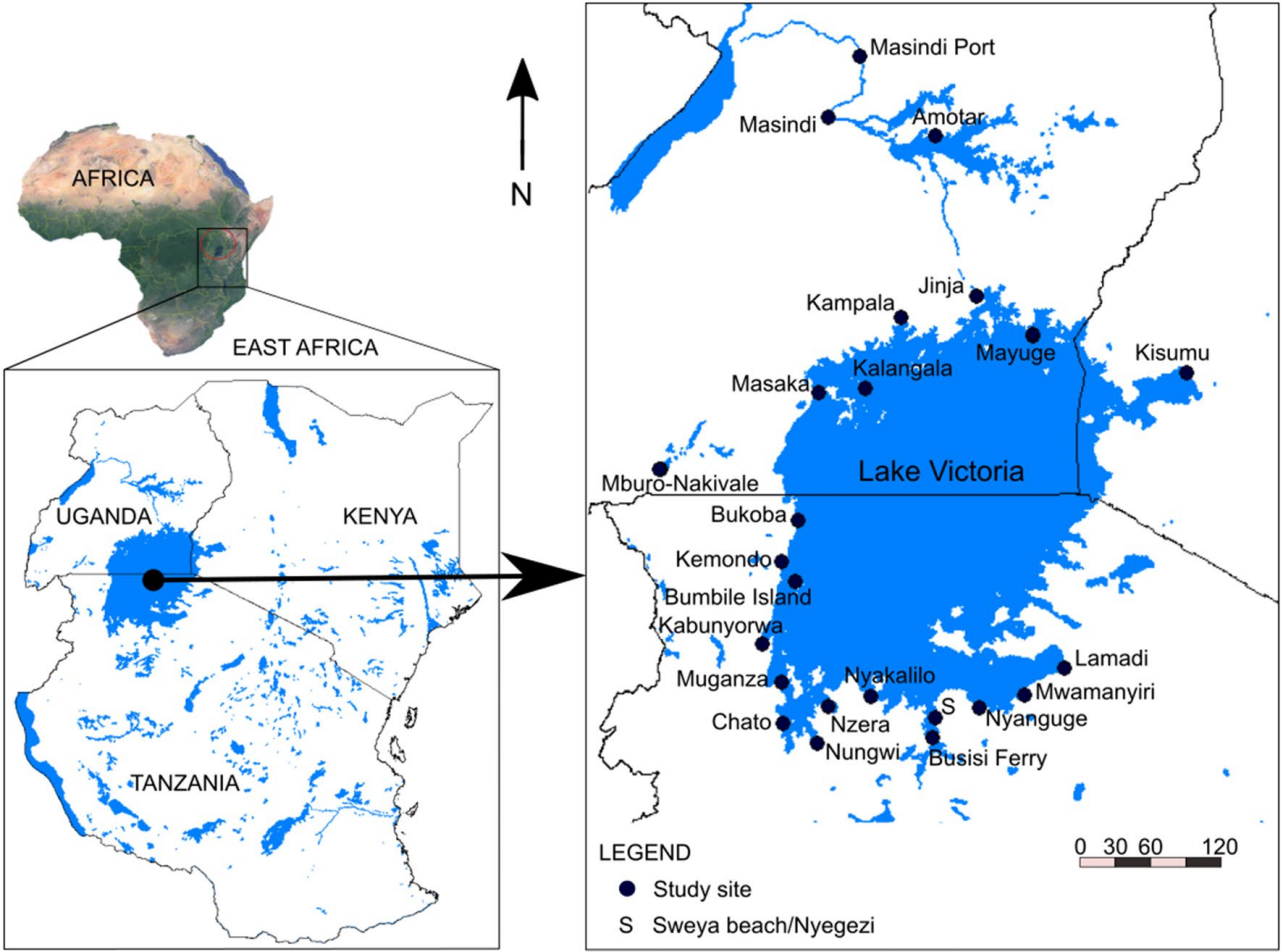
Study area map of East African countries that share Lake Victoria and the sampling sites for this study are shown

Pulmonate snails of the genus *Bulinus* were collected from 20 locations around Lake Victoria and (for comparative purposes) from an additional four locations in the neighbouring aquatic systems of the River Nile and Lake Mburo-Nakivale (Fig. 1, Table 1). Sampling was carried out in open waters, on shoreline banks, around islands and in bordering marsh habitats where water was either stagnant or relatively calm. Specimens were hand-picked off water plants, rocks, stones or the floor bottom where they were more easily accessible or collected with strainers, long handheld scoops and dredges in more challenging situations (e.g. deeper waters). Dredging was carried out repeatedly per site in depths from 2 m down to approximately 25 m in the Kenyan and Ugandan part of the lake. In most of the sites, sampling was carried out close to active anthropogenic activities (e.g. fish landing sites or ferry docks) and for at least 30–60 min. All specimens were collected during various field trips from 2010–2019 and snails identified as *Bulinus* spp. were preserved in 80% ethanol.

**Table 1:** Locality, voucher, sequence and haplotype information for the *Bulinus* spp. from Lake Victoria studied

Species	Locality	Country	Latitude	Longitude	Voucher No.	Sequence ID	Habitat	Haplotype ID	GenBank ID	
									*cox*1	ITS2
*B. truncatus*	Igabiro	Tanzania	− 1.17769	31.87792	UGSB 22907	Bkt26885	Stone and rocks	BKT1	MT707360	MT707212
*B. truncatus*	Igabiro	Tanzania	− 1.17769	31.87792	UGSB 22908	BKt26886	Stone and rocks	BKT2	MT707361	
*B. truncatus*	Kemondo	Tanzania	− 1.47796	31.7498	UGSB 22909	Ket26887	Stone and rocks	KET1	MT707362	MT707222
*B. truncatus*	Kemondo	Tanzania	− 1.47796	31.7498	UGSB 22910	Ket26888	Stone and rocks	KET2	MT707363	
*Bulinus* sp. 2	Bumbire Island	Tanzania	− 1.61476	31.85625	UGSB 22911	Bit26889	Island	BIT1	MT707364	
*Bulinus* sp. 2	Bumbire Island	Tanzania	− 1.61476	31.85625	UGSB 22912	Bit26890	Island	BIT2	MT707365	MT707234
*Bulinus* sp. 2	Bumbire Island	Tanzania	− 1.61476	31.85625	UGSB 22946	Bit26924	Island	BIT3	MT707366	
*Bulinus* sp. 2	Bumbire Island	Tanzania	− 1.61476	31.85625	UGSB 23464	Bit27073	Island	BIT4	MT707367	
*Bulinus* sp. 2	Bumbire Island	Tanzania	− 1.61476	31.85625	UGSB 23465	Bit27074	Island	BIT5	MT707368	
*Bulinus* sp. 2	Kabunyorwa	Tanzania	− 2.06018	31.61382	UGSB 22913	Kbt26891	Papyrus	KBT1	MT707369	
*Bulinus* sp. 2	Kabunyorwa	Tanzania	− 2.06018	31.61382	UGSB 22914	Kbt26892	Papyrus	KBT2	MT707370	MT707235
*B. truncatus*	Muganza	Tanzania	− 2.33702	31.75166	UGSB 22915	Mut26893	Open water	MUT1	MT707371	
*B. truncatus*	Muganza	Tanzania	− 2.33702	31.75166	UGSB 22916	Mut26894	Open water	MUT2	MT707372	MT707220
*Bulinus* sp. 2	Muganza	Tanzania	− 2.33702	31.75166	UGSB 22940	Mut26918	Water Hyasin	MUT3	MT707373	MT707236
*Bulinus* sp. 2	Muganza	Tanzania	− 2.33702	31.75166	UGSB 22941	Mut26919	Water Hyasin	MUT4	MT707374	
*B. truncatus*	Muganza	Tanzania	-2.33702	31.75166	UGSB 22945	Mut26923	Open water	MUT5	MT707375	
*Bulinus sp. 2*	Chato	Tanzania	− 2.63292	31.76368	UGSB 23463	Cht27072	Papyrus	CHT1	MT707376	
*Bulinus* sp. 2	Nungwe	Tanzania	− 2.77446	32.0136	UGSB 22919	Nut26897	Papyrus	NUT1	MT707377	
*Bulinus* sp. 2	Nungwe	Tanzania	− 2.77446	32.0136	UGSB 22920	Nut26898	Papyrus	NUT2	MT707378	MT707233
*Bulinus* sp. 2	Nungwi	Tanzania	− 2.77446	32.0136	UGSB 23466	Nut27075	Papyrus	NUT3	MT707379	
*B. tropicus*	Nzera	Tanzania	− 2.51209	32.09845	UGSB 22921	Nzt26899	Sand beach	NZT1	MT707380	
*B. tropicus*	Nzera	Tanzania	− 2.51209	32.09845	UGSB 22922	Nzt26900	Sand beach	NZT2	MT707381	MT707229
*B. truncatus*	Nyakalilo	Tanzania	− 2.43669	32.41158	UGSB 22923	Nyt26901	Stone beach	NYT1	MT707382	MT707221
*Bulinus* sp. 2	Nyakalilo	Tanzania	− 2.43669	32.41158	UGSB 22924	Nyt26902	Papyrus	NYT2	MT707383	MT707237
*Bulinus* sp. 2	Busisi	Tanzania	− 2.72626	32.87034	UGSB 22925	But26903	Water Hyasin	BUT1	MT707384	MT707230
*Bulinus* sp. 2	Busisi	Tanzania	− 2.72626	32.87034	UGSB 22926	But26904	Water Hyasin	BUT2	MT707385	
*Bulinus* sp. 2	Nyegezi A	Tanzania	− 2.585	32.88541	UGSB 22927	Sat26905	Water Hyasin	SAT1	MT707386	MT707238
*B. truncatus*	Nyegezi A	Tanzania	− 2.585	32.88541	UGSB 22928	Sat26906	Stone and rocks	SAT2	MT707387	MT707223
*B. truncatus*	Nyegezi B	Tanzania	− 2.58434	32.88331	UGSB 22929	Sbt26907	Stone and rocks	SBT1	MT707387	MT707216
*B. truncatus*	Nyegezi B	Tanzania	− 2.58434	32.88331	UGSB 22930	Sbt26908	Stone and rocks	SBT2	MT707387	
*B. tropicus*	Nyegezi C	Tanzania	− 2.58388	33.51714	UGSB 22942	Sct26920	Sand beach	SCT1	MT707390	MT707228
*Bulinus* sp. 1	Nyegezi C	Tanzania	− 2.58388	33.51714	UGSB 22943	Sct26921	Sand beach	SCT2	MT707391	
*Bulinus* sp. 1	Nyegezi C	Tanzania	− 2.58388	33.51714	UGSB 22944	Sct26922	Sand beach	SCT3	MT707392	MT707226
*Bulinus* sp. 2	Nyanguge	Tanzania	− 2.51911	33.20884	UGSB 22933	Ngt26911	Marshes/papyrus	NGT1	MT707393	
*Bulinus* sp. 2	Nyanguge	Tanzania	− 2.51911	33.20884	UGSB 22934	Ngt26912	Marshes/papyrus	NGT2	MT707394	MT707232
*Bulinus* sp. 2	Nyanguge	Tanzania	− 2.51911	33.20884	UGSB 23467	Ngt27076	Marshes/papyrus	NGT3	MT707395	
*Bulinus* sp. 2	Mwamanyiri	Tanzania	− 2.43022	33.5424	UGSB 22935	Mwt26913	Marshes/papyrus	MWT1	MT707396	MT707231
*Bulinus* sp. 2	Mwamanyiri	Tanzania	− 2.43022	33.5424	UGSB 22936	Mwt26914	Marshes/papyrus	MWT2	MT707397	
*Bulinus* sp. 2	Mwamanyiri	Tanzania	− 2.43022	33.5424	UGSB 23468	Mwt27077	Marshes/papyrus	MWT3	MT707398	
*Bulinus* sp. 2	Lamadi	Tanzania	− 2.23738	33.84236	UGSB 22937	Lat26915	Marshes/papyrus	LAT1	MT707399	
*Bulinus* sp. 2	Lamadi	Tanzania	− 2.23738	33.84236	UGSB 22938	Lat26916	Marshes/papyrus	LAT2	MT707400	MT707240
*Bulinus* sp. 2	Lamadi	Tanzania	− 2.23738	33.84236	UGSB 23469	Lat27078	Marshes/papyrus	LAT4	MT707401	
*Bulinus* sp. 2	Kisumu	Kenya	− 0.12739	34.74232	UGSB 23446	Kik27055	Water Hyasin	KIK1	MT707402	
*Bulinus* sp. 2	Kisumu	Kenya	− 0.12739	34.74232	UGSB 23447	Kik27056	Water Hyasin	KIK2	MT707403	MT707239
*Bulinus* sp. 2	Kisumu	Kenya	− 0.12739	34.74232	UGSB 23448	kik27057	Water Hyasin	KIK3	MT707406	
*B. truncatus*	Nile	Uganda	0.42084	33.19639	UGSB 23452	Niu27061	Open water	JIU1	MT707407	MT707214
*B. truncatus*	Mayuge	Uganda	0.14067	33.60258	UGSB 16758	Myu22513	Open water	MYU1	MT707404	
*B. truncatus*	Mayuge	Uganda	0.14067	33.60258	UGSB 16757	Myu22514	Open water	MYU2	MT707405	
*B. truncatus*	Mayuge	Uganda	0.14067	33.60258	UGSB 23453	Myu27062	Open water	MYU3	MT707408	
*B. truncatus*	Mayuge	Uganda	0.14067	33.60258	UGSB 23454	Myu27063	Open water	MYU4	MT707409	
*B. truncatus*	Mayuge	Uganda	0.14067	33.60258	UGSB 23603	Myu27108	Open water	MYU5	MT707411	
*B. truncatus*	Mayuge	Uganda	0.14067	33.60258	UGSB 23604	Myu27109	Open water	MYU6	MT707410	MT707218
*B. truncatus*	Masindi	Uganda	2.12852	32.32919	UGSB 23457	Msu27066	Nile river	MSU1	MT707412	
*B. truncatus*	Masindi	Uganda	2.12852	32.32919	UGSB 23458	Msu27067	Nile river	MSU2	MT707413	
*B. truncatus*	Masindi	Uganda	2.12852	32.32919	UGSB 23459	Msu27068	Nile river	MSU3	MT707414	
*B. truncatus*	Masindi	Uganda	2.12852	32.32919	UGSB 23460	Msu27069	Nile river	MSU4	MT707415	MT707217
*B. truncatus*	Masindi	Uganda	2.12852	32.32919	UGSB 23607	Msu27112	Nile river	MSU5	MT707416	
*B. truncatus*	Masindi Port	Uganda	1.69249	32.09664	UGSB 16759	Mpu22515	Nile river	MPU1	MT707417	
*B. truncatus*	Masindi Port	Uganda	1.69249	32.09664	UGSB 16760	Mpu22516	Nile river	MPU2	MT707418	
*B. truncatus*	Masindi Port	Uganda	1.69249	32.09664	UGSB 23610	Mpu27115	Nile river	MPU3	MT707419	MT707219
*Bulinus* sp. 2	Masaka	Uganda	− 0.27263	32.02691	UGSB 23461	Mku27070	Water Hyasin	MKU1	MT707420	
*Bulinus* sp. 2	Kampala	Uganda	− 0.27263	32.02691	UGSB 23596	Kau27101	Water Hyasin	KAU1	MT707421	MT707241
*B. truncatus*	Kampala	Uganda	− 0.27263	32.02691	UGSB 23598	Kau27103	Open water	KAU2	MT707422	MT707215
*Bulinus* sp. 2	Kampala	Uganda	− 0.27263	32.02691	UGSB 23599	Kau27104	Water Hyasin	KAU3	MT707423	
*Bulinus* sp. 2	Amotar	Uganda	1.55822	32.88828	UGSB 23613	Amu27118	Marshes/papyrus	AMU1	MT707424	
*B. truncatus*	Kalangala	Uganda	0.30371	32.28927	UGSB 16774	Klu22530	Open water	KLU1	MT707425	
*B. truncatus*	Kalangala	Uganda	0.30371	32.28927	UGSB 23616	Klu27121	Open water	KLU2	MT707426	MT707213
*B. truncatus*	Lake Mburo	Uganda	− 0.638	30.9528	UG3	BspJDUG3		MNU1	MT707427	
*B. truncatus*	River Rwizi	Uganda	− 0.6863	30.8856	UG19	BspJUG19		MNU2	MT707428	
*B. truncatus*	River Rwizi	Uganda	− 0.6863	30.8856	UG22	BspJUG22		MNU3	MT707429	
*B. truncatus*	Lake Mburo	Uganda	− 0.6951	30.8514	UG27	BspJUG27		MNU4	MT707430	
*B. truncatus*	Lake Nakivale	Uganda	− 0.8205	30.8559	UG7	BspJDUG7		MNU5	MT707431	
*Bulinus* sp. 2	Lake Nakivale	Uganda	− 0.8205	30.8559	UG98	BspJUG98		MNU6	MT707432	
*B. forskalii*	Lake Nakivale	Uganda	− 0.8205	30.8559	UG76	BspJUG76		MNU7	MT707403	
*B. truncatus*	Lake Albert	Uganda			A1	HQ121558		LAU1	HQ121558	
*B. truncatus*	Lake Albert	Uganda			A2	HQ121559		LAU2	HQ121559	
*B. truncatus*	Lake Albert	Uganda			A3	HQ121560		LAU3	HQ121560	
*B. truncatus*	Katosho swamp	Tanzania			T1	HQ121562		KST1	HQ121562	
*B. truncatus*	Lake Albert	Uganda			BO (Booma)	GU176747		LAU4	GU176747	
*B. truncatus*	Lake Albert	Uganda			1PD (Piida)	GU176748		LAU5	GU176748	
*B. truncatus*	Lake Albert	Uganda			TO (Toonya)	GU176749		LAU6	GU176749	
*B. truncatus*	Nyanguge	Tanzania			Nyanguge	AM286313		NGT	AM286313	
*Bulinus* sp. 2	Kisumu	Kenya			ADC farm	AM286297		AFK1	AM286297	
*B. truncatus*	Lake Sagara	Tanzania			T04em43A	AM286298		LST	AM286298	

*Abbreviation*: UGSB, University of Giessen Systematics and Biodiversity collection

### DNA extraction, amplification and sequencing

Genomic DNA was extracted using the CTAB method [38] from 2–5 specimens per locality for a total of 74 specimens. A 655-bp target fragment of the mtDNA *cox*1 gene was amplified using primers and PCR conditions given by Folmer et al. [39]. In a few cases, the region was amplified using the primers LCO1490 [39] and COR722B [40] and PCR conditions as detailed by Kane et al. [37]. Primers LT1 and ITS2-RIXO and PRC conditions stated by Almeyda-Artigas et al. [41] and Bargues et al. [42] were used to amplify the rDNA ITS2 region. Sanger sequencing was performed by LGC Genomics GmbH (Berlin, Germany).

### Phylogenetic analyses

Chromatograms were assembled and inspected using Geneious version 8.0.6 (Biomatters, Auckland, New Zealand; Kearse et al. [43]). Multiple alignments were generated for each marker, with the ClustalW tool [44] implemented in BioEdit version 7.0.5.3 [45]. Newly generated sequences from 74 specimens were combined with 57 additional available sequence data from GenBank to expand our datasets (Additional file 1: Table S1). The online program MAFFT [46], was used to align the ITS2 partition. The phylogenetic trees of the concatenated datasets of 620 bp *cox*1 and ITS2 were estimated using Maximum Likelihood (ML) and Bayesian Inference (BI) analyses. The *cox*1 and ITS2 partitions were concatenated using Sequences Matrix version 1.2.8 [47]. In both cases, *Indoplanorbis exustus* was used as the outgroup. The best sequence evolutionary model to each partition was evaluated with jModelTest version 2.1.4 [48]. Based on the Akaikeʼs information criterion (AIC), HYK + G and GTR+G were selected as the best evolutionary models for *cox*1 and ITS2 datasets, respectively. ML analysis was conducted using Randomized Accelerated Maximum Likelihood (RAxML version 7.0.4; [49]) with a bootstrap of 1000 replicates. Bayesian inference analysis, to obtain an ultrametric tree for the General Mixed Yule Coalescent (GMYC) model of species delimitation [50], was carried out using BEAST version 1.8.4 [51]. Runs consisted of 5,000,000 MCMC generations, sampling every 500th tree. Validation of convergence and mixing was assessed in Tracer 1.5 [52] to ensure that all effective sample size (ESS) values were > 200. We used TreeAnnotator 1.8.4 (BEAST package) to identify the maximum clade credibility (MCC) tree by discarding 50% of the trees as ‘burn-in’.

We applied two DNA-based species delimitation methods (SDMs) with single and multiple delimiting thresholds to resolve the species boundaries in *Bulinus* specimens incorporated. These were the Poisson Tree Process (PTP [53]) and the GMYC method as mentioned above. Both mPTP (maximum likelihood, PTP and Bayesian, bPTP) and GMYC analyses were carried out with the web-based service at https://species.h-its.org/.

### Phylogeographical and population analyses

Phylogeographical analyses were performed for the novel *cox*1 sequences of the *Bulinus* specimens from Lake Victoria and the neighbouring systems (i.e. Lake Mburo-Nakivale and the River Nile). The dataset consisted of the 74 sequences generated herein. The relationships between haplotypes were identified through a statistical parsimony network constructed in TCS version 1.21 [54] with 95% confidence.

For genetic diversity, differentiation and population expansion or shrinkage *cox*1 sequences belonging to the *Bulinus* specimens from Lake Victoria basin were split into two groups representing *B. truncatus* and *Bulinus* sp. 2. *Bulinus truncatus* sequences were divided into three subpopulations based on habitat, namely, lentic sand substrate, lentic stones and rock substrates and lotic habitats. The sequences forming the *Bulinus* sp. 2 group were also divided into three subpopulations based on lentic habitats; islands, papyrus swamps and marshes (water hyacinth). We estimated haplotype diversity (*h*) and nucleotide diversity (*π*) [55] using DnaSP version 6.12.03 [56]. Moreover, we performed analyses of molecular variance (AMOVA), to examine the amount of genetic variability within and between populations, using Arlequin version 3.5.2.2 [57].

The mitochondrial DNA sequence data were also tested for deviation from neutral expectations (e.g. population expansion events). Genetic equilibrium was assessed using Arlequin version 3.5.2.2 [57] by calculating Tajima’s *D* [58] and Fu’s *Fs* [59]. Under the assumption of selective neutrality, Arlequin version 3.5.2.2 was also used for mismatch distribution analysis of pairwise differences within and between populations. The relative population sizes (θ_0_ and θ_1_) and relative time since population expansion (τ) were estimated also using Arlequin version 3.5.2.2. The estimated τ value was used to estimate time since expansion using the formula τ= 2 µt, where µ is the mutation rate per site per generation and τ is the time since population expansion [60]. In the present study, the substitution rate of 1.22 ± 0.27% per million years was applied for the mtDNA (*cox*1) region [61]. Additionally, a Mantel test for matrix correspondence between genetic and geographical distances was performed using GenAlEx version 6.5. [62] to test the isolation by distance (IBD). The input matrices for genetic distance were constructed in Mega X [63].

## Results

### Species identification and phylogenetic relationships

**Fig. 2:**
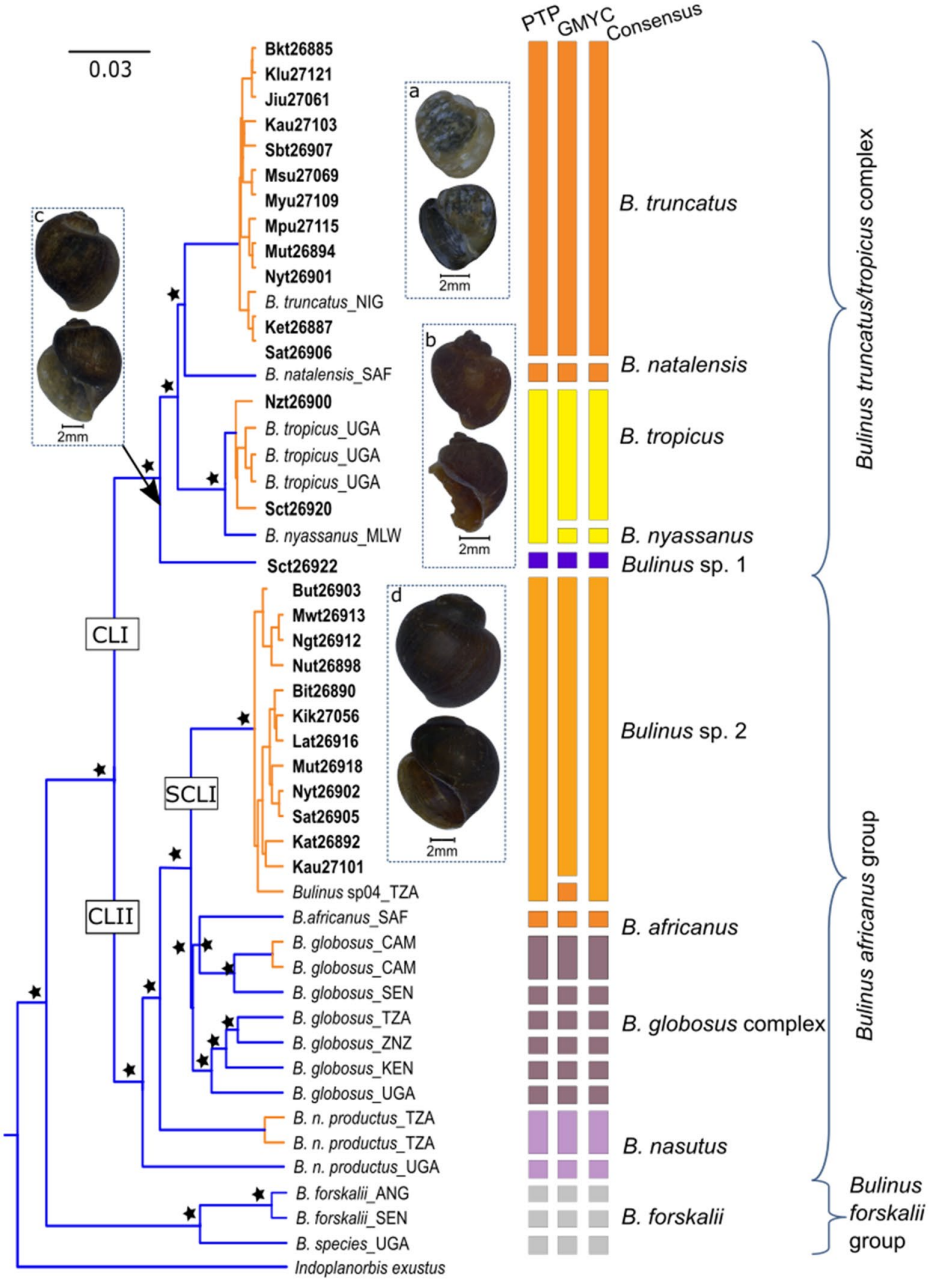
The BI phylogenetic tree of *Bulinus* species with bars, on the right, denoting different species delimitation results, based on the dataset of concatenated *cox*1 and ITS2 sequences. Within the phylogeny, nodes supported and shared between BI and ML methods are marked with stars where support equates to 90–100% (ML) and 0.95–1 (BI). Names in bold are for specimens collected in the present study and the rest have been retrieved from GenBank: *B. truncatus* (**a**); *B. tropicus* (**b**); *Bulinus* sp. 1 (**c**); *Bulinus* sp. 2 (**d**). Locality details are provided in Table 1. *Abbreviations*: CLI, Clade I; CLII, Clade II; SCI, Subclade I; SCII, Subclade II. The three-letter abbreviation for countries is also given. *Notes*: the blue colour represents different species, while green stands for the same species according to species delimitation methods. The three-letter abbreviations represent countries: NIG, Nigeria; SAF, South Africa; UGA, Uganda; MLW, Malawi; TZA, Tanzania; CAM, Cameroon; SEN, Senegal; ZNZ, Zanzibar; KEN, Kenya; ANG, Angola. The information for sequences retrieved from the GenBank is presented in Additional file 1: Table S1

Both Maximum Likelihood (ML) and Bayesian Inference (BI) analyses of concatenated genes (*cox*1 and ITS2) generated strongly supported phylogenies that revealed the presence of two main *Bulinus* groups in Lake Victoria (Fig. 2). Clade I comprised of *B. truncatus/tropicus* complex and Clade II contained the *B. africanus* group Moreover, Clade I exhibited a complex structure that corresponded to *Bulinus* specimens that inhabited open waters and sandy beaches of Lake Victoria. For instance, specimen labelled Sct26922, collected from Nyegezi on the Tanzanian side of the lake, was found in shallow waters near sandy beaches coexisting with a physid species. Both species delimitation methods (SDMs), PTP and GMYC, categorised the specimen as a unique molecular operational taxonomic unit (MOTU; *Bulinus* sp. 1.). Clade I also contained *Bulinus* samples collected outside the lake albeit within the lake basin i.e. the Lake Mburo-Nakivale and Nile River ecosystems, denoting that these species are not endemic to Lake Victoria. Moreover, combined phylogenetic and SDMs analyses revealed the presence of *B. truncatus* and *B. tropicus* in Lake Victoria, although *B. truncatus* are more widely distributed than *B. tropicus*.

Novel sequences forming subclade I (SCI, Fig. 2) were isolated from *Bulinus* specimens collected from the banks and marshes surrounding the lake and small islands, particularly Bumbire in Tanzania and Mayuge in Uganda. Although these *Bulinus* specimens formed a well-defined and supported clade in both analyses (ML = 100% and BI = 1.0), they did not intermingle with other species within the clade; they formed a definite group of their own (*Bulinus* sp. 2). As shown in Fig. 2, despite the complexity or the presence of cryptic species in GenBank sequences designated as *B. globosus*, SDMs treated *Bulinus* specimens from the banks and surrounding marshes, regardless of the location they were collected, as one species (MOTU). The specimens from the banks matched only with *Bulinus* sp. T04em43A (GenBank: AM286298) from Lake Sagara in Tanzania, and accordingly, SDMs placed them under the same MOTU. The phylogeny and SDMs from *cox*1 also identified *Bulinus nasutus productus* and *B. forskalii* collected from Lake Mburo-Nakivale system within the Lake Victoria basin. Similar results are shown for *cox*1 analyses (Additional file 2: Figure S1)

### Phylogeographical and population analyses

**Fig. 3:**
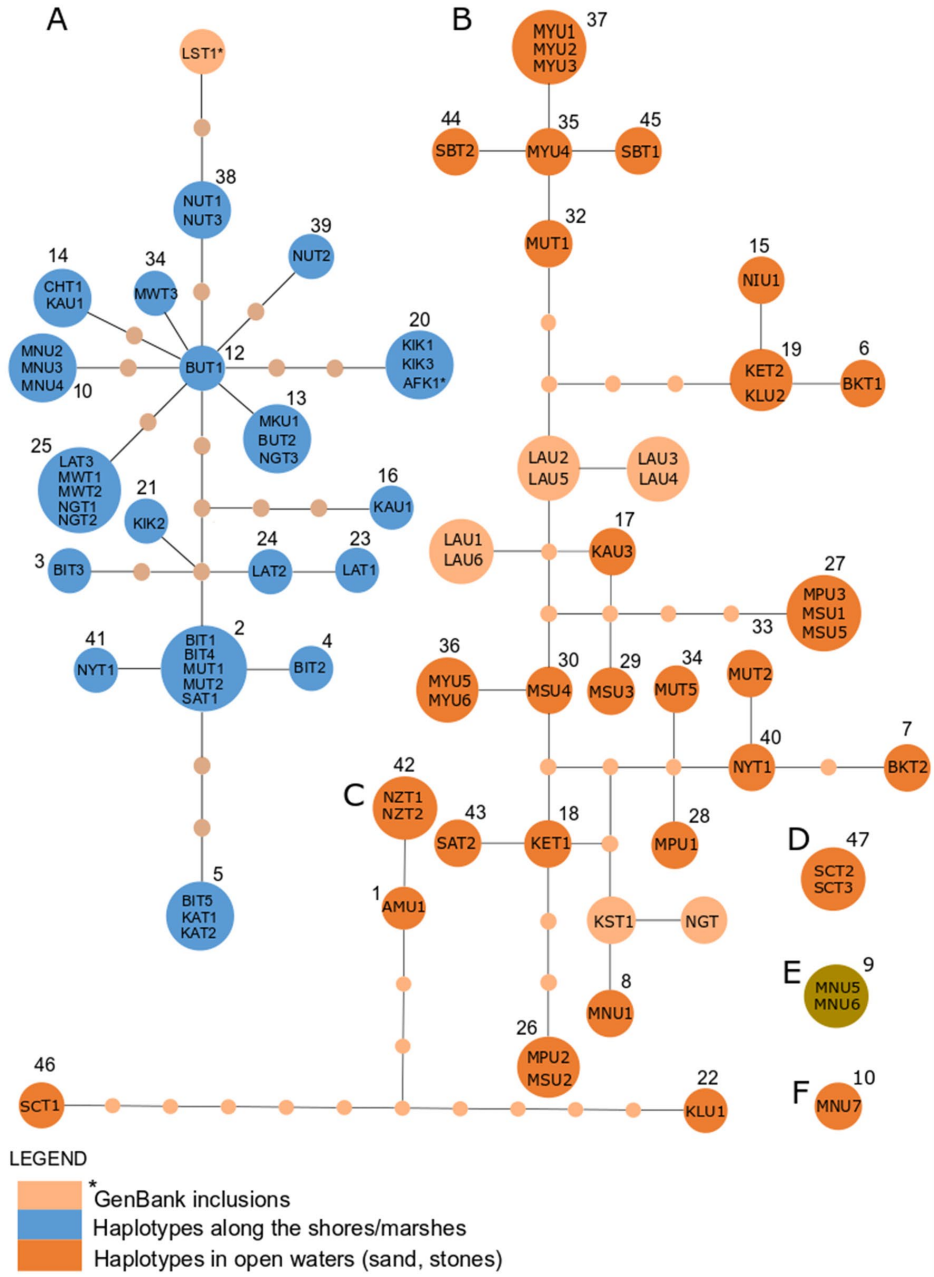
Statistical parsimony network of *cox*1 sequences (connecting limit: 95%) of *Bulinus* species from Lake Victoria; *Bulinus* sp. 2 (**a**), *B. truncatus* (**b**), *B. tropicus* (**c**), *Bulinus* sp. 1 (**d**), *B. nasutus* (**e**) and *B. forskalii* (**f**). The size of the circles corresponds to the number of individuals belonging to the respective haplotype. Mutational steps for the missing haplotypes are presented as small circles, and numbers correspond to the number of individuals with a given haplotype. Green stands for GenBank material

**Fig. 4:**
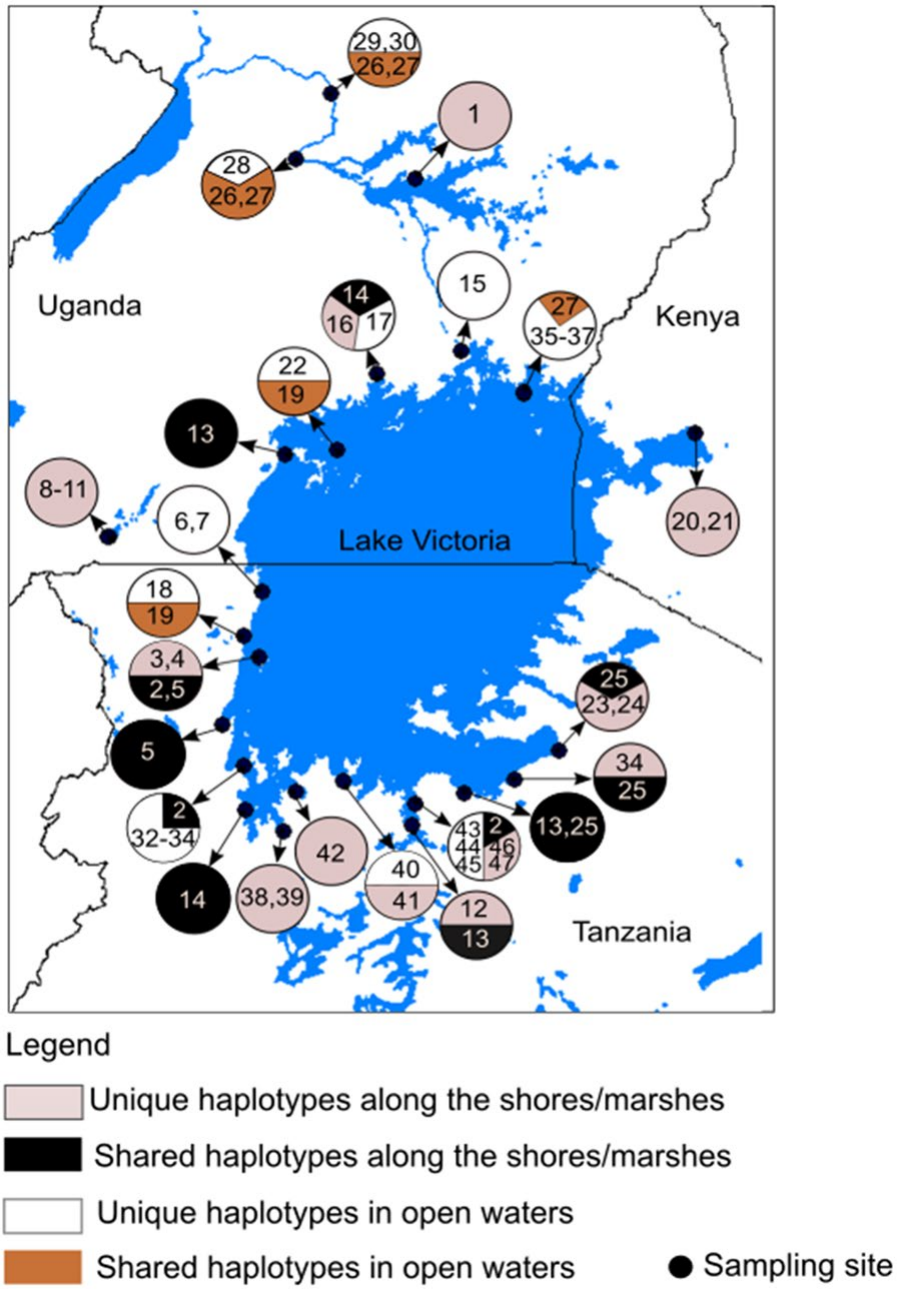
A map of Lake Victoria showing *Bulinus* species haplotypes distribution. Specimen details are provided in Table 1

Although the phylogeographical analysis of the present study did not acquire sufficient samples from the Kenyan side and small islands, in particular, TCS networks supported the phylogenies (Fig. 3) that Lake Victoria is dominated by two distinct clades of *Bulinus* species; species occurring in the lake proper and those inhabiting the banks and surrounding marshes. However, at the confidence limit of 0.95, the dataset comprising specimens from the banks and marshes represented *B. africanus* group species (A in Fig. 3) while those from the open water (lake proper) revealed three separate networks: *B. truncatus*/*B. tropicus* complex, i.e. *B. truncatus* (B in Fig. 3), *B. tropicus* (C in Fig. 3) and an undefined species *Bulinus* sp. 1 (D in Fig. 3). *Bulinus nasutus productus* and *B. forskalii* from Lakes Mburo-Nakivale systems formed separate networks (E and F in Fig. 3, respectively). Similar to phylogeny and SDMs, TCS analysis revealed a *Bulinus* specimen collected from Nyegezi in Tanzania (Haplotype 47) as a distinct species (D in Fig. 3). Generally, the TCS analysis showed that *Bulinus* species had shared haplotypes distributed throughout Lake Victoria, indicating that these species are not localised in the lake (Fig. 4). Moreover, the TCS analysis corroborated phylogenies and SDMs that specimens sampled from the banks and surrounding marshes of Lake Victoria relate to potentially undescribed bulinid species, *Bulinus* sp. T04em43A (GenBank: AM286298) from Lake Sagara, Tanzania and *Bulinus* sp. K3.03 (GenBank: AM286297) from Lake Victoria in Kisumu, Kenya.

The mtDNA loci showed high overall haplotype (*h*) and nucleotide (*π*) diversity among populations (0.984 and 0.071). The population analysis of *B. truncatus* revealed 22 haplotypes, out of which 2 haplotypes were shared between sand beaches and river systems (Table 2). On the other hand, *Bulinus* sp. 2 (Clade II) population consisted of 17 haplotypes and a least one haplotype was shared between two habitats i.e. islands, papyrus and water hyacinths. Nevertheless, no haplotype was shared among the three habitats; an indication that haplotypes are specific to habitats. Nucleotide and haplotype diversities were also high within each habitat (Table 2).

**Table 2:** Results of genetic diversities, AMOVAs and mismatch distribution for populations of *Bulinus* spp. in Lake Victoria

	*Bulinus truncatus*				*Bulinu*s sp. 2			
	Mean (*n* = 29)	Lentic sand substrate (*n* = 14)	Lentic stone & rocks substrate (*n* = 8)	River systems (*n* = 7)	Mean (*n* = 31)	Islands (*n* = 5)	Swamp papyrus (*n* = 15)	Marshes water hyacinth (*n* = 11)
Haplotype (h)	22	11	8	5	17	4	10	7
Haplotype diversity (*h*)	0.978 ± 0.005	0.956 ± 0.0156	1.000 ± 0.022	0.905 ± 0.040	0.940 ± 0.005	0.900 ± 0.016	0.895 ± 0.022	0.909 ± 0.041
Nucleotide diversity (*π*)	0.01067	0.01134	0.01014	0.08387	0.00753	0.005	0.010	0.084
*F*_*ST*_ (*P*-value)	0.034 (0.045)	0.014 (0.297)	0.048 (0.072)	0.047 (0.027)	0.064 (0.020)	0.077 (0.081)	− 0.016 (0.432)	0.080 (0.000)
Tajima’s *D* (*P*-value)	− 0.126 (0.484)	− 1.092 **(**0.144)	0.096 (0.575)	0.618 (0.733)	− 1.158 (0.112)	− 1.162 **(**0.058)	− 0.606 (0.279)	− 0.317 (0.414)
Fu’s *FS* (*P*-value)	− 1.735 (0.237)	− 2.549 (0.097)	− 3.273 (0.022)	0.617 (0.585)	− 5.439 (0.014)	− 0.445 (0.277)	− 2.088 (0.149)	0.644 (0.312)

*Note*: The ordering of specimens was based on the habitats they were found

The inbreeding coefficients (*F*_*ST*_), defined from the AMOVA, for *B. truncatus* and *Bulinus* sp. 2 populations were 0.034 (*P* = 0.045) and 0.064 (*P* = 0.020) respectively. These *F*_*ST*_ values demonstrate an apparently low genetic differentiation between habitats. Table 2 summarises the genetic variations of the *Bulinus* in these groups occurring in Lake Victoria. Generally, *F*_*ST*_ values (0.021–0.023) between and within habitats groups were low (Table 2) indicating that the gene flow among *Bulinus* species populations and subpopulations within the Lake Victoria is high. The AMOVA concurs with the haplotype network, in which there was no clear demarcation between the localities where a given specimen was collected and its genetic affiliation with other haplotypes (Figs. 3, 4).

The estimates of Tajima’s *D* and Fu’s *F*s test of *Bulinus* populations from Lake Victoria (i.e. within the lake, banks and surrounding marshes) were negative and statistically significant (Table 2), which denotes that the *Bulinus* species in the lake have undergone a recent population expansion. With a 95% confidence interval (CI), estimates of θ_0_ and θ_1_ for *Bulinus* species indicated that populations expanded, both demographically and spatially, from a compact to a considerable size (Table 3). Using the tau values (τ) of 3.787 and 4 for the *B. truncatus* in the open water and *Bulinus* sp. 2 occurring in the banks and marshes of Lake Victoria, we roughly estimate the starting time for *Bulinus* rapid population expansion to be between 207,694 (± 107,823) and 464,678 (± 278,312) years ago (Table 3).

**Table 3:** Results of the mismatch distribution analyses for the demographic and spatial expansions of the *Bulinus* species from Lake Victoria populations and time since expansion

	*Bulinus truncatus*						*Bulinus* sp. 2					
	Demographic expansion			Spatial expansion			Demographic expansion			Spatial expansion		
	Lentic sand substrate	Lentic stones & rock substrates	Lotic habitats	Lentic sand substrate	Lentic stones & rock substrates	Lotic habitats	Islands	Swamp papyrus	Marshes^a^	Islands	Swamp papyrus	Marshes^a^
SSD (P-value)	0.095 (0.280)	0.079 (< 0.0001)	0.024 (0.160)	0.095 (0.430)	0.069 (0.040)	0.020 (0.600)	0.023 (0.130)	0.087 (0.010)	0.069 (0.170)	0.021 (0.370)	0.059 (0.100)	0.063 (0.410)
RI (P-value)	0.310 (0.280)	0.219 (< 0.0001)	0.073 (0.280)	0.310 (0.510)	0.219 (0.110)	0.073 (0.640)	0.032 (0.450)	0.092 (0.580)	0.213 (0.200)	0.032 (0.680)	0.092 (0.430)	0.213 (0.390)
Theta0/Theta	0.000	1.900	0.000	0.05589	0.001	0.001	0.000	5.500	1.622	0.010	0.010	1.180
Theta1	16.211	3414.978	34.961				46.951	3414.978	49.882			
τ (Cl)	3.469 (2.258-6.211)	4.000 (2.822–7.725)	5.556 (2.994–7.803)	3.233 (1.555-6.146)	5.811 (2.309–7.509)	5.363 (2.788–7.639)	6.277 (3.646–8.352)	5.000 (3.555–13.682)	5.438 (3.344–10.984)	6.121 (3.805–7.578)	7.234 (2.901–8.282)	5.453 (2.619–10.173)
T in years	222,824	256,951	356,946	207,694	373,288	344,497	403,243	321,188	349,293	393,184	464,678	350,287
ΔT in years	± 77,788	± 75,655	± 164,610	± 107,823	± 224,936	± 165,400	± 169,000	± 92,843	± 134,498	± 148,784	± 278,312	± 182,033

*Abbreviations*: SSD, sum of squared deviations; RI, raggedness index; CI, 95% confidence interval; τ, population parameter Tau; T, time since expansion

^a^Water hyacinth

There was no significant correlation between genetic and geographical distances within the *Bulinus* population (*B. truncatus*) inhabiting the proper lake (*r*^2^ = 0.018, *P* > 0.05) or those (*Bulinus* sp. 2) from the banks, islands and marshes (*r*^2^ = 0.0038, *P* > 0.05). Overall all *Bulinus* samples from Lake Victoria did not exhibit any correlation between genetic variations and distance (*r*^2^ = 0.0175, *P* > 0.05), indicating the variation in genetic distance is mainly due to taxonomic differences as already shown by both phylogeny and parsimony networks.

## Discussion

### Identity of *Bulinus* in Lake Victoria and their phylogenetic affinities

The present study, to our knowledge, is the first to apply molecular techniques on the longitudinally surveyed *Bulinus* species occurring in Lake Victoria. A majority of studies on molluscs in Lake Victoria have been conducted on *Biomphalaria* species for their role in the spread of intestinal schistosomiasis [13, 18, 25]. The present study provides molecular-based evidence on the presence of two *Bulinus* groups in the lake; *B. truncatus/B. tropicus* occupying the open waters, covering sand beaches, stones and submerged rocks, while *B. africanus* group dominates the banks, small islands and surrounding marshes. Although the number of species determined by PTP and GMYC was slightly indecisive, the present study supports previous findings [27, 31, 35–37] that molecular methods could delineate the monophyletic subclade comprising of *B. truncatus* and its sibling *B. tropicus* (Fig. 2), which are morphologically difficult to distinguish [17].

From Mandahl-Barth [64] to present, the taxonomy of *Bulinus* species in Lake Victoria is in scrutiny. According to Brown [17], four species of *Bulinus* occur in Lake Victoria and the most common are the coexisting diploid and tetraploid populations forming the *B. truncatus/B. tropicus* complex that lack an apparent taxonomic boundary. Other *Bulinus* material was classified as *B. trigonus* and *B. transversalis* [64], though Brown [17] suggested that they might be lacustrine morphs of *B. tropicus* and *B. truncatus*. However, the present molecular analysis of material from Bumbire Island, the type-locality for *B. transversalis* [17], grouped the material with *Bulinus* sp. 2, which is regarded by the present study as *B. ugandae*. Nonetheless, the specimens from the island were smaller than those collected from the banks and marshes elsewhere. Although potentially topotypic material was collected and a single species only occurred there, we cannot conclude that *Bulinus* sp. 2 is, in fact, *B. transversalis*. Morphological characteristics of the snails studied here suggest that nowadays the waters around the island are rather inhabited by *B. ugandae*.

Phylogenetic analysis accompanied by SDMs also revealed a unique MOTU of *Bulinus*, *Bulinus* sp. 2, in Lake Victoria (Fig. 2, Clade II/Subclade I), which was strongly supported as sister to *B. globosus* in the *B. africanus* group. Although our phylogenetic analyses did not find sequences of *Bulinus* from Lake Victoria in the GenBank database to compare with, our sampling is reasonable to relate the *Bulinus* sp. 2 to *B. ugandae*. In our perusal of the literature regarding genus *Bulinus* in Lake Victoria, only *B. ugandae* shares similar features to the present material. Both Mandahl-Barth [29] and Brown [17] while scrutinising the morphological characters of *B. ugandae*, they questioned its taxonomic position in relation to *B. globosus*. Loker et al. [65] also acknowledged the challenging task of separating accurately *B. globosus* and *B. ugandae* from the Lake Victoria region. Moreover, Mwambungu [32] encountered *B. ugandae* in the Speke Gulf of the lake on the Tanzanian side and Ngupula & Kayanda [33] found *B. ugandae* and *B. transversalis* in Uganda. A study Opisa et al. [34] also found *B. globosus* distributed along the shores of Lake Victoria in Kisumu, Kenya. The close relatedness of the present specimen and *Bulinus* sp. (GenBank: AM286297) from Kisumu in Kenya [26] further shows a wide distribution of *B. ugandae* in Lake Victoria. While the separation of *B. ugandae* from *B. globosus* morphologically is paradoxical [17, 29], most workers used the names interchangeably.

In this analysis, we also found a unique MOTU of *Bulinus* (*Bulinus* sp. 1) which were collected in the southern part of Lake Victoria at Sweya beach in Nyegezi, Mwanza. The strong phylogenetic support for *Bulinus* sp. 1 (BS = 100%, PP = 1.00; Fig. 2) within the *B. truncatus/B. tropicus* clade and the separation of *B. truncatus* and *B. tropicus* haplotypic networks (Fig. 3), is a clear indication that *Bulinus* sp. 1 is a different species. The closest match to the *cox*1 sequences of *Bulinus* sp. 1 was 97.22% with *B. tropicus* (GenBank: KJ157492) from Cameroon [36]. Morphologically, *Bulinus* sp. 1 were similar to other members of the *B. truncatus/B. tropicus* species complex except that they were found co-existing with *B. tropicus* and physids in much shallower water on the mud-covered sand beach. Given that this species is neither *B. truncatus* nor *B*. *tropicus* nor *B. transversalis* (see above), we remain with *B. trigonus* as the sole known member of the *B. truncatus/B. tropicus* complex for Lake Victoria. More research is, however, needed to decide whether *Bulinus* sp. 1 indeed represents *B. trigonus*.

It is noteworthy that the shallow lake systems west of Lake Victoria harbour at least two different *Bulinus* species (i.e. *B. nasutus productus* and *B. forskalii*; see Figs. 2, 3, 4). Summarizing the current *Bulinus* diversity (Table 4), the Lake Victoria fauna consists of at least four species: *B. truncatus*; *B. tropicus*; *Bulinus* sp. 1 (*B. trigonus*?); and *B. ugandae* (*Bulinus* sp. 2).

**Table 4:** Species diversity of the genus *Bulinus* in the Lake Victoria basin

Species	Occurrence	Role as host	Reference	Present study
*B. africanus*	Near LV in Kenya, Mwanza, Tanzania	Main host in South Africa, NW Tanzania	Brown [17]	Not found
*B. globosus*	Mwanza, LV, Kisumu	Southern Africa, Main host in NW Tanzania	Loker et al [65]; Opisa et al [34]	Not found
*B. forskalii*	LV	not confirmed	Brown [17]	*B. forskalii* (not found in lake proper)
*B. nasutus productus*	Eastern shore LV	Main host in NW Tanzania	Brown [17] Mandahl-Barth [14]	*B. nasutus productus* (not found in lake proper)
*B. tropicus*	Not mentioned before	Not known		*B. tropicus*
*B. reticulatus*	Near Kisumu and Mwanza	Not known	Brown [17]; Loker et al [65]	Not found
*B. trigonus*	LV and Lake Edward	*B. truncatus*: main host in NE, W and N Africa	Brown [17]	*B. trigonus?*
*B. transversalis*	LV and Victoria Nile	Not known	Brown [17]; Mandahl-Barth [14]	Not found
*B. ugandae*	LV, NW Tanzania	Not known	Brown [17]; Mandahl-Barth [14]	*B. ugandae*

*Notes*: Taxa mentioned in the literature, their distribution, assumed or proven roles as intermediate hosts for *S. haematobium* are provided. Where possible, findings from the recent study are compared to the previous information

*Abbreviation*: LV, Lake Victoria

### Genetic population analysis

The genetic variation, analysis of molecular variance (AMOVA), and isolation by distance showed *Bulinus* species populations in Lake Victoria to be panmictic. The overall F_*ST*_ value (0.034) in *cox*1 was significantly low, which may be explained by high gene flow rates among *Bulinus* populations in Lake Victoria to favour the evolution of phenotypic plasticity within species [66]. Also, AMOVA produced F_*ST*_ values within populations ranging from 0.00–0.080, meaning *Bulinus* species in Lake Victoria consist of overlapping populations. However, the ranges of genetic differentiation between populations (0.00–0.08) are comparable to previous studies on *Bulinus* species [67–69], who attributed the variations to self-fertilization within the populations. Given the size of the lake and high gene flow observed, it can be hypothesized that *Bulinus* species in Lake Victoria could be both cross and self-fertilizers. The cross-fertilization and pathogenesis in the banks and surrounding marshes may be increased due to intrusion of water weeds water hyacinth (*Eichhornia crassipes*), which are implicated in creating new habitats for snails [70, 71]. Moreover, our findings corroborate Standley et al. [13] who argued about the impossibility of sudden demographical events that would influence the genetic diversity and population structure of snail populations in Lake Victoria.

Studies in Lake Victoria have shown that, despite its large size, it is one of the youngest large lakes in the African Rift and has existed only 400,000 years ago with three complete desiccations in between, and the current water body was refilled about 14,600 years ago [15]. In contrast, our findings showed the *Bulinus* populations in Lake Victoria began spatial and demographic expansion about 99,700–743,000 years before the present. The explanation may be twofold, (i) the snails colonized the lake from neighbouring aquatic systems during the last refilling and (ii) the lake did not completely dry to reflect the 100,000 years of Milankovitch climate forcing cycles [10, 15]. Both scenarios could be associated with the low levels of genetic variation and population structure indices at the intrapopulation level within the *Bulinus* species in Lake Victoria [29]. Our results, however, support the scenario that the current biota in Lake Victoria recolonized the refilling lake from refugia as argued by Nalugwa et al. [72] given that about 100,000 years ago Lake Victoria probably collected its waters from regions near Lake Tanganyika [10]. The occurrence of *Bulinus* species in Lake Sagara in the Ugalla-Malagarasi drainage system in western Tanzania [37] and *B. truncatus* in Lakes Kivu and Tanganyika (Katosho swamp) [73], respectively, similar to those found in Lake Victoria, further supports the invasion theory.

### Ecological aspects

Lake Victoria experienced tremendous ecological perturbations in the Anthropocene, and human activities nowadays might contribute significantly to the mixing of populations across the lake and adjacent aquatic ecosystems [74]. Even though we found no indication of such human effects for the *Bulinus* populations studied, future studies employing more sensitive markers should focus on these potentially confounding factors affecting population structures across the lake. Differential impacts of human disturbances on snail existence and abundances have been demonstrated in the Kenyan part of Lake Victoria [75]. Whereas some species might disappear, others, including intermediate host snails, i.e. pulmonates generally, might be even favoured by eutrophication processes and as such might increase the risks of transmission [75, 76]. The general abundance of pulmonate snails is high throughout the lake and marsh systems (FC and CA, personal observations). This in concert with reduced predator pressure from molluscivorous fishes might account for the comparatively high biomasses of certain gastropod species including some of the *Bulinus* spp. There is evidence for the roles of habitats in shaping (eco-)morphotypes in the less diverse *Biomphalaria* in Lake Victoria [77]. Such effects remain to be studied in detail for *Bulinus*, although our results so far indicated a link between habitat types and genetic diversity.

### Parasitological implications of *Bulinus* species in Lake Victoria

Lake Victoria is one of the most well-known hotspots of schistosomiasis worldwide with fishing communities and school-aged children reported to be the most infected demographic groups in the surrounding countries of Kenya, Tanzania and Uganda [18–20, 23, 24]. However, a vast majority of reports on schistosomiasis in the lake and banks have focused on *Biomphalaria* species and their consequential *S. mansoni* [13, 18, 25]. There are two specific or subspecific forms of *Biomphalaria* species that preserve transmission of schistosomiasis in the lake: (i) *B. sudanica*, mainly found along the shores and surrounding marshes and swamps; and (ii) *B. choanomphala*, a more in-depth water inhabitant of Lake Victoria (Stanley et al. [18], but see Zhang et al. [25] for a discussion on species identified). The present findings showed that two dominant taxa of *Bulinus* occur in the lake: (i) *B. ugandae* (*Bulinus* sp. 2), mainly found along the banks and surrounding marshes and swamps in the mainland and islands; and (ii) members of *B. truncatus/B. tropicus* complex, which are found in open water habitats.

Although the present study did not test the collected snails for patent and prepatent infections with *Schistosoma* spp. or other digenean trematodes, the presence of certain *Bulinus* species in Lake Victoria potentially implies the presence of *S. haematobium*. Both *B. truncatus/B. tropicus* complex, *B. africanus* and *B. forskalii* group members have already been implicated in the transmission of *S. haematobium* elsewhere in Africa [17, 31, 78]. *Bulinus nasutus productus* has been known to occur around the eastern shore of the lake [33] and was now also found in the west. This species has been shown to be involved in *S. haematobium* transmission [12]. Even if *B. tropicus* is not known to be an intermediate host for *Schistosoma* species [17], the present findings are particularly important because hitherto the morphological distinction within *B. truncatus/B. tropicus* complex is challenging [17]. *Bulinus truncatus* is not yet known to be a host in equatorial Africa; however, there is potential [17] since it is the main host in the regions up the Nile river (Nile Province of South Sudan) where high prevalences of *S. haematobium* infections have been reported [79]. *Bulinus ugandae* is apparently not known to host *S. haematobium* but screening for *B. globosus* should continue in and around Lake Victoria. Given that *B. africanus* group members are found close by (*B. nasustus* and *B. forskalii* in satellite lakes that are hydrologically connected to Lake Victoria), there is a hidden risk for the prevalence of *S. haematobium*. Therefore, the occurrence and wide distribution of *Bulinus* species in Lake Victoria potentially threaten the health of communities living along the shores and on islands of the lake who depend on the lake for their livelihood. This situation is even triggered by the increasing pollution of the lake, which has recently been demonstrated to worsen the infection risks [80], this is yet another factor complicating the combat of schistosomiasis in this hotspot [24]. Future studies should undertake more experimental approaches to snail transmission. Another promising tool in predicting and identifying transmission potential (contamination and exposure) is the environmental DNA approach [81]. This has very recently been successfully used for environmental surveillance of schistosomiasis [82].

Previous studies on the prevalence of *S. mansoni* and *S. haematobium* showed the species were partitioned according to distance from the lake, i.e. *S. mansoni* occurred close to the lake and *S. haematobium* further on the hinterland [83]. Additionally, the spatial distribution of *S. haematobium* was in line with the presence of streams and ponds [79]. These observations imply that intermediate host species of *Biomphalaria* and *Bulinus*, the respective intermediate hosts for *S. mansoni* and *S. haematobium*, likely occur inside and outside the lake, respectively [18]. Our results, on the other hand, corroborate the previous observations that arrange of *Bulinus* species are present in the lake and are confirmed here to be widespread, but their role in *S. haematobium* transmission remains uncertain. A widely neglected aspect relates to schistosomiasis as a disease of veterinary concern [27]. *Bulinus tropicus* and *B. ugandae* are a well-known host for *S. bovis*, a parasite extensively infecting livestock [81]. Zoonotic schistosomiasis is currently largely underestimated [84] but could be studied in the setting of Lake Victoria in the future. Zoonotic schistosomiasis could be of high concern for both livestock and also wildlife existing in the adjacent world-famous national parks.

## Conclusions

This study has reported two major *Bulinus* groups and at least four species occurring in Lake Victoria, *B. truncatus/B. tropicus* complex and *B. africanus* inhabiting vegetation-free sand and stone beaches, and banks and surrounding marshes/papyrus beds on the mainland and islands. These findings reflect previous findings on *Biomphalaria* species. Since in this study, we did not trace how far deep *B. truncatus/B. tropicus* complex can occur, we recommend a depth abundance relationship analysis for *Bulinus* species be carried out. Our findings also conclude that the assumed *B. ugandae* dominates the banks and surrounding marshes. *Bulinus trigonus* might indeed be a separate species whereas the *B. transversalis* remains to be studied genetically. Following our findings, a parasitological examination of *Bulinus* species around the lake is paramount to understanding their role in the epidemiology of urogenital schistosomiasis and its subsequential control. It is also recommended to study in parallel patterns in co-occurring *Biomphalaria* spp. throughout seasonal cycles and along environmental gradients.

## Supplementary information

**Additional file 1: Table S1.** Summary of additional sequence data from the crater lakes and other regions retrieved from GenBank with the localities and haplotypes noted. Additionally, the locality, voucher, sequence and haplotype information for the *Bulinus* species from Lake Victoria studied for the first time herein are also given. *Abbreviation*: UGSB, University of Giessen Systematics and Biodiversity.

**Additional file 2: Figure S1.** The BI phylogenetic tree of *Bulinus* species with bars, on the right, denoting different species delimitation results, based on the dataset of concatenated *cox*1 sequences. Within the phylogeny, nodes supported and shared between BI and ML methods are marked with stars where support equates to 90–100% (ML) and 0.95–1 (BI). Names in bold denote specimens collected in the present study and the rest have been retrieved from the GenBank. Locality details are provided in Table 1. Blue colour represents different species, while green represents the same species as resolved by species delimitation methods. The information for sequences retrieved from the GenBank is presented in Additional file 1: Table S1. *Abbreviations*: SC1, Subclade 1; SC2, Subclade 2; SC3, Subclade 3; SC4, Subclade 4. The three-letter abbreviations represent countries: NIG, Nigeria; SAF, South Africa; UGA, Uganda; MLW, Malawi; TZA, Tanzania; CAM, Cameroon; SEN, Senegal; ZNZ, Zanzibar; KEN, Kenya; ANG, Angola; EGY, EGYPT; DRC, Democratic Republic of Congo.

## Data Availability

All data generated or analysed in the course of this study are included in the article, its additional files or have been deposited in the University of Giessen Systematics and Biodiversity (UGSB) repository, which are available upon request. Additionally, newly generated sequences were deposited in the GenBank database under the accession numbers MT707360-MT707433 (*cox*1) and MT707212-MT707241 (ITS2).

## Abbreviations

SDMs: Species delimitation methods
PTP: Poisson tree processes
GMYC: Generalized mixed Yule coalescent
AMOVA: Analysis of molecular variance
ML: Maximum likelihood
BI: Bayesian inference
NCBI: The National Center for Biotechnology Information
