# Supplementary figures and images for: Assessing the diversity and distribution of potential intermediate hosts snails for urogenital schistosomiasis: Bulinus spp. (Gastropoda: Planorbidae) of Lake Victoria
Source: Parasit Vectors. 2020 Aug 14;13:418. doi: 10.1186/s13071-020-04281-1 (PMC7427762; doi:10.1186/s13071-020-04281-1)

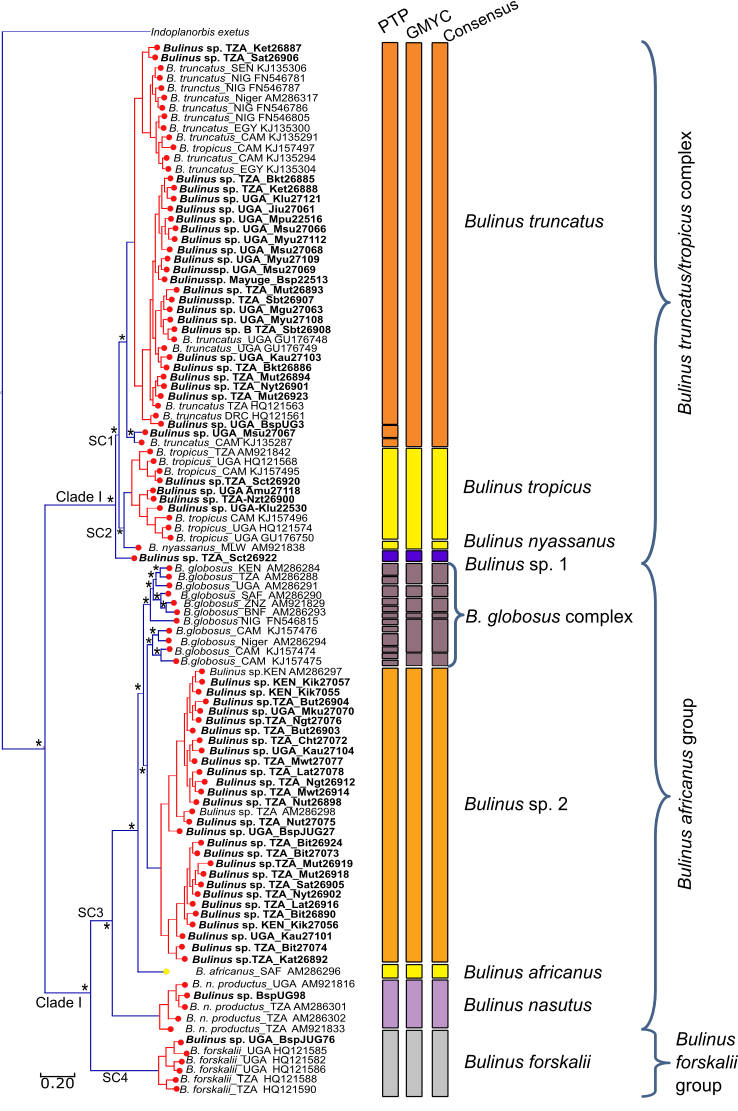

Supplement: Supplementary file 2 — Additional file 2: Figure S1. The BI phylogenetic tree of Bulinus species with bars, on the right, denoting different species delimitation results, based on the dataset of concatenated cox1 sequences. Within the phylogeny, nodes supported and shared between BI and ML methods are marked with stars where support equates to 90–100% (ML) and 0.95–1 (BI). Names in bold denote specimens collected in the present study and the rest have been retrieved from the GenBank. Locality details are provided in Table 1. Blue colour represents different species, while green represents the same species as resolved by species delimitation methods. The information for sequences retrieved from the GenBank is presented in Additional file 1: Table S1. Abbreviations: SC1, Subclade 1; SC2, Subclade 2; SC3, Subclade 3; SC4, Subclade 4. The three-letter abbreviations represent countries: NIG, Nigeria; SAF, South Africa; UGA, Uganda; MLW, Malawi; TZA, Tanzania; CAM, Cameroon; SEN, Senegal; ZNZ, Zanzibar; KEN, Kenya; ANG, Angola; EGY, EGYPT; DRC, Democratic Republic of Congo. [file 13071_2020_4281_MOESM2_ESM.png]
